# Supplementary material for: The mediodorsal pulvinar coordinates the macaque fronto-parietal network during rhythmic spatial attention
Source: Nat Commun. 2019 Jan 15;10:215. doi: 10.1038/s41467-018-08151-4 (PMC6333835; doi:10.1038/s41467-018-08151-4)
Supplement: Supplementary file 1 — Supplementary Information [file 41467_2018_8151_MOESM1_ESM.pdf]

Supplementary Materials

The mediodorsal pulvinar coordinates the macaque fronto-parietal network during rhythmic spatial attention

Fiebelkorn et al.

SUPPLEMENTAL MATERIALS

ROI	all	visual	visual-movement	movement
**mdPul**	224	20	19	13
**FEF**	238	36	45	17
**LIP**	259	39	41	18

Supplementary Table 1. *Numbers of neurons in each ROI with significantly increased task-related responses.* Neurons were classified as visual (i.e., only visual-sensory activity), visual-movement (i.e., both visual-sensory and saccade-related activity), and movement (i.e., only saccade-related activity) types.

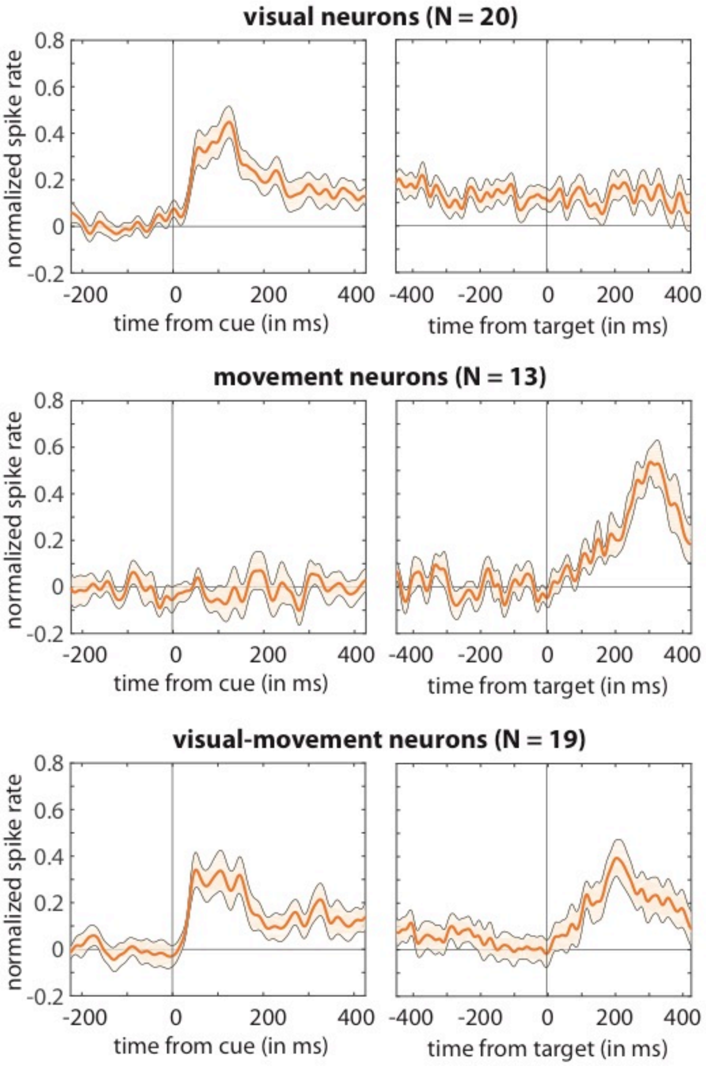

Supplementary Figure 1. *Normalized population spike rates in mdPul by cell type.* Significant spiking during the cue-target delay was observed in visual and visual-movement neurons, but not in movement neurons.

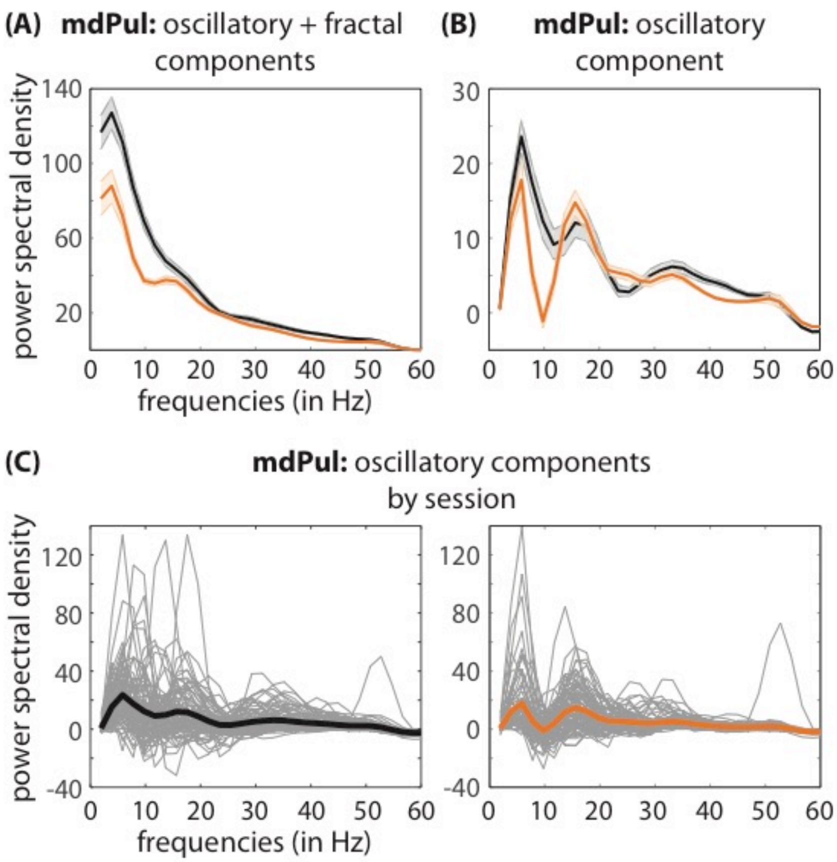

Supplementary Figure 2. *Power spectral density in mdPul when response fields overlapped the cued location, prior to the cue (black line) and during the cue-target delay (orange line).* **(A)** There was a general drop in low-frequency power following the cue, but **(B)** removing the fractal (or 1/f) component, using the IRASA approach (Wen & Liu, 2016), demonstrates an apparent oscillatory peak in the theta range during both trial periods, as well as a peak in the alpha/low-beta range. **(C)** The oscillatory components for each recording session, prior to the cue (on left) and during the cue-target delay (on right), relative to the average across sessions (bolded lines).

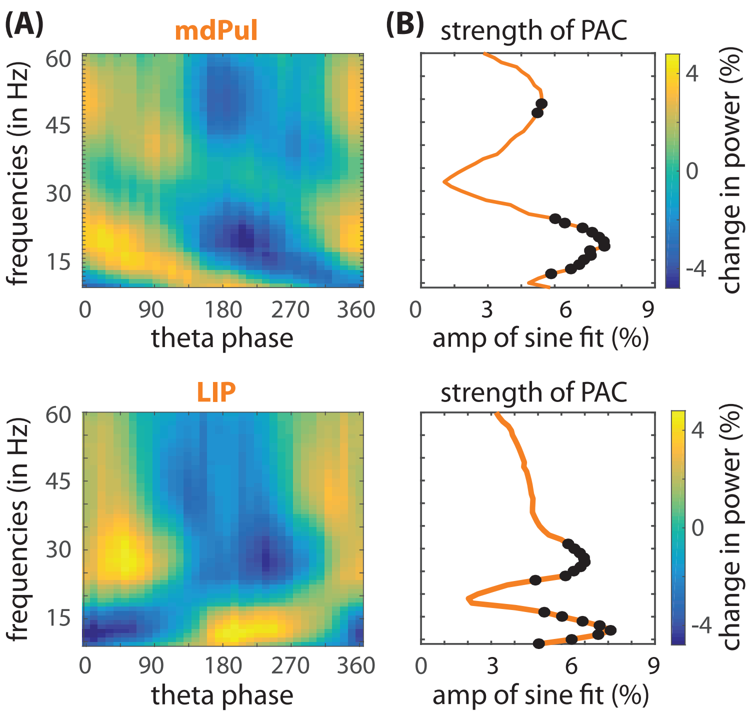

Supplementary Figure 3. *Phase-amplitude coupling (PAC) between theta phase and oscillatory power (from 9–60 Hz) in MdPul and LIP during spatial attention.* **(A)** For mdPul**,** higher alpha/low-beta power occurred during the theta phase associated with relatively better visual-target detection (i.e., the "good" theta phase). For LIP**,** higher alpha/low-beta power occurred during the theta phase associated with relatively worse visual-target detection (i.e., the "poor" theta phase) [4]. **(B)** Oscillatory power as a function of theta phase was fit with one-cycle sine waves, with the amplitude of those fitted sine waves measuring the strength of PAC (see Fig. 2A for a depiction of a similar approach). The black dots represent statistically significant PAC after corrections for multiple comparisons.

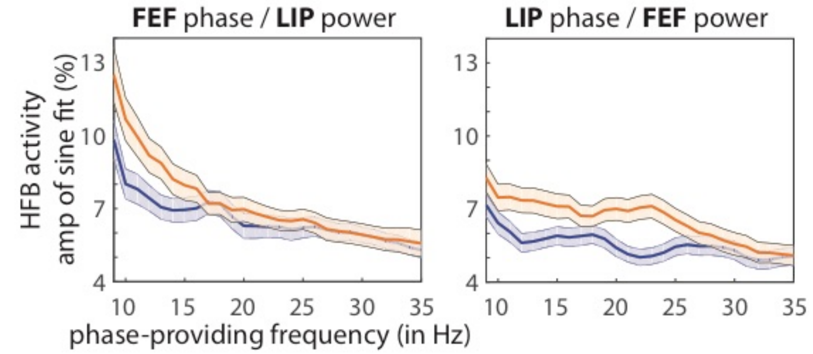

Supplementary Figure 4. *Phase-amplitude coupling (PAC) between alpha/low-beta phase in FEF (or LIP) and high-frequency band (HFB) power in LIP (or FEF) increases during spatial attention.* HFB power, a proxy for population spiking, was binned by oscillatory phase (at frequencies from 9–35 Hz). PAC was measured by fitting one-cycle sine waves to the resulting HFB by phase functions (see Fig. 2A for depiction of a similar approach) [4]. The amplitudes of the fitted sine waves was used to estimate the strength of PAC at each phase-providing frequency. The above plots compare PAC when receptive/response fields overlapped either the cued (orange) or the non-cued (blue) location. These results demonsrate that alpha/low-beta activity is functionally relevant in FEF and LIP, organizing between-region interactions under conditions of spatial attention. Shaded regions around the lines represent SEs.

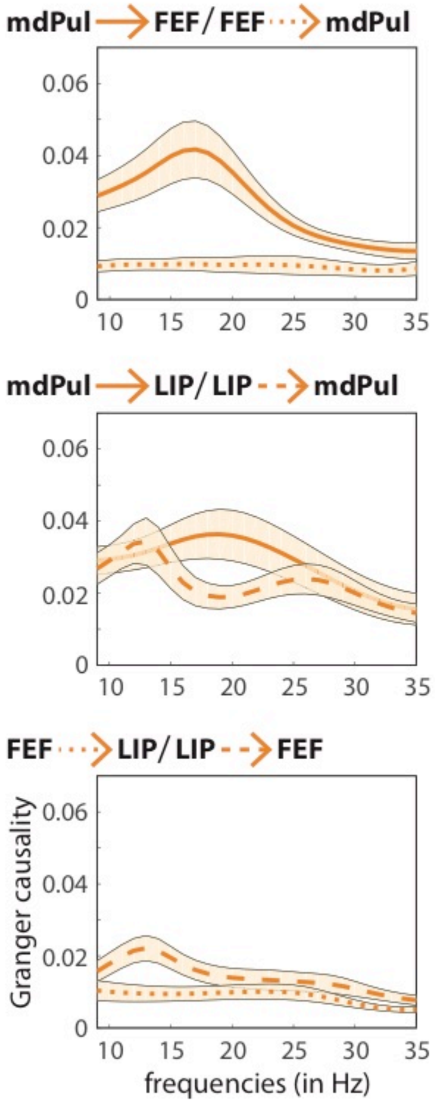

Supplementary Figure 5. *Conditional Granger causality for a subset of recording sessions (N = 31) when all 3 ROIs had overlapping response fields.* These results are similar to those reported in Figure 5.

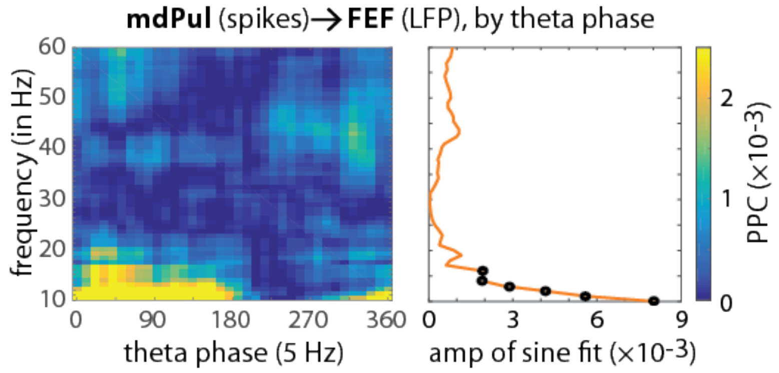

Supplementary Figure 6. *Spikes in mdPul also seem to be specifically coupled to alpha/low-beta activity in FEF during the "good" theta phase.* Spike-LFP phase coupling (from 9–60 Hz) was calculated in overlapping theta-phase bins (on left), using step sizes of 10 degrees. The resulting functions were then fit with one-cycle sine waves. The amplitude of these sine waves provided a measure of how strongly spike-LFP phase coupling was modulated by the phase of theta rhythms (on right, see Fig. 2A for depiction of a similar approach). The black dots represent statistically signficant results after corrections for multiple comparisons. See Fig. 4B for additional evidence.

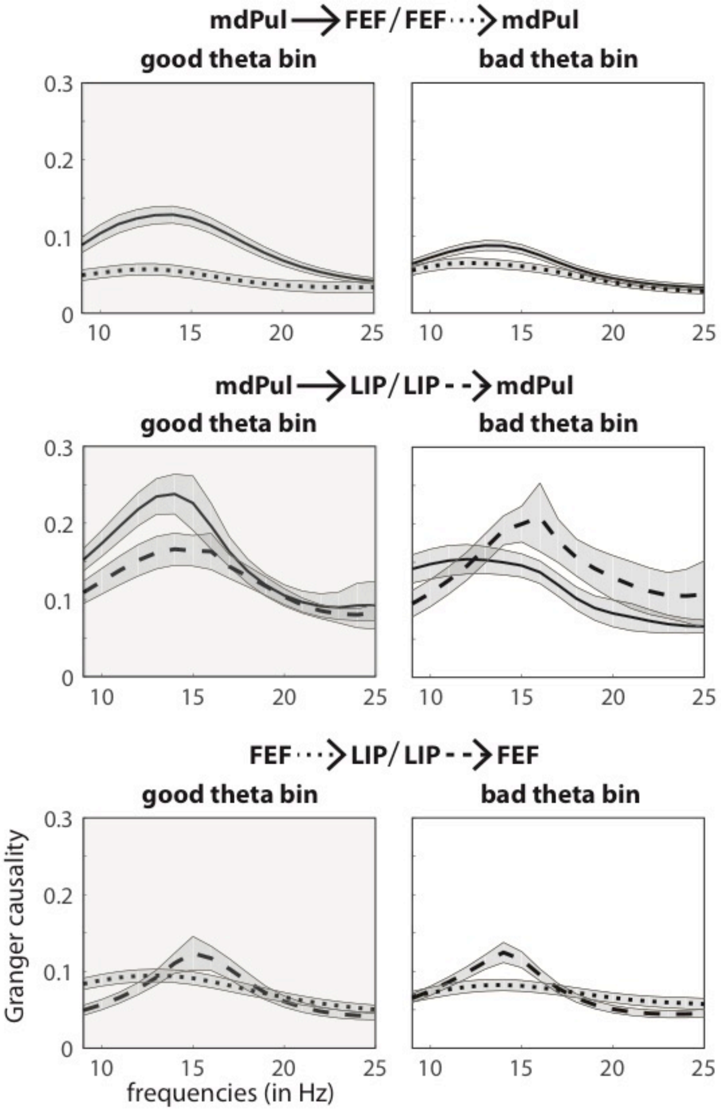

Supplementary Figure 7. *Granger causal influence indicates that mdPul regulates alpha/low-beta activity in cortical hubs of the attention network (i.e., FEF and LIP).* Shows Granger causal influence after binning based on theta phase ("good" vs. "poor"). Here, we first used a stratification procedure to equate alpha/low-beta power, both across ROIs and across theta-phase bins. These findings confirm the results presented in Figure 4, demonstrating that mdPul specifically regulates cortical activity during periods of relatively better visual-target detection (i.e., during the "good" theta phase). Shaded regions around the lines represent SEs.

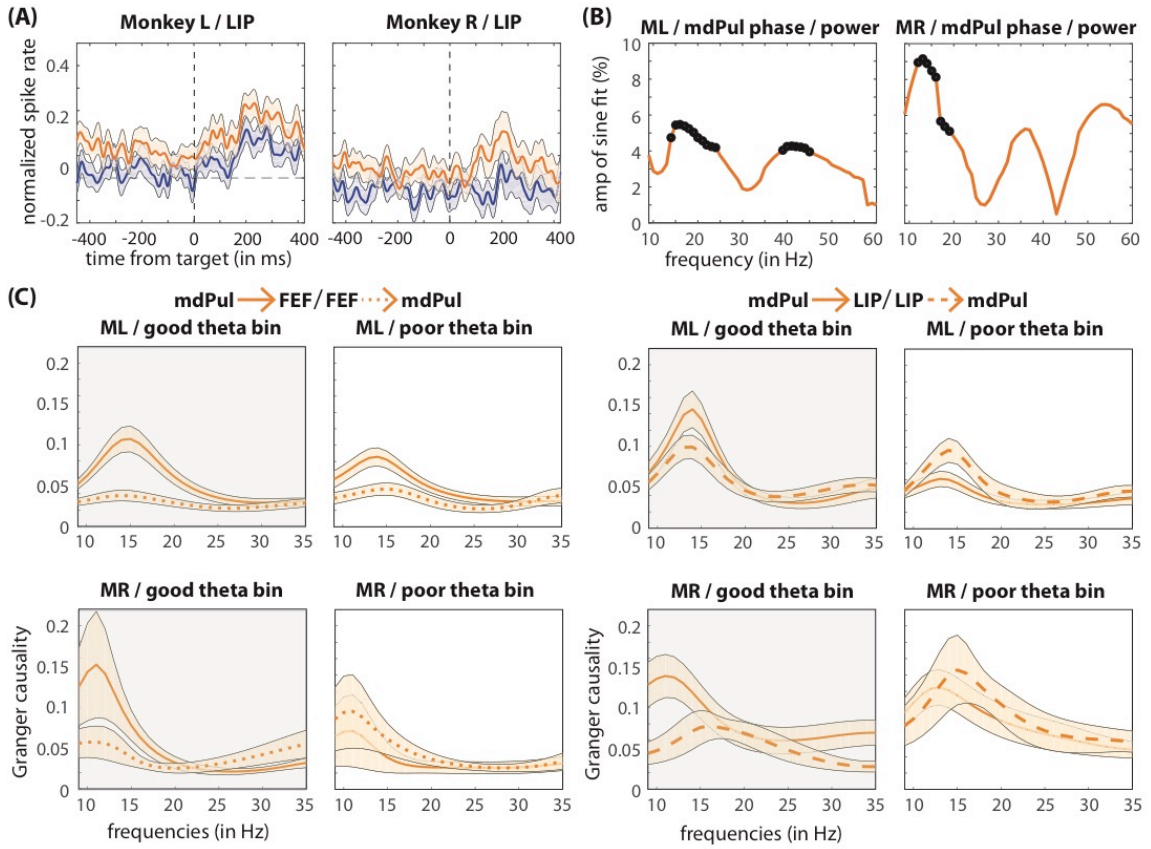

Supplementary Figure 8. *Both monkeys (L and R) contributed to effects that we observed after combining data across the two animals.* For example, **(A)** shows greater delay spiking when receptive fields overlapped the cued location (in orange), relative to when receptive fields overlapped the non-cued location (in blue), **(B)** shows significant coupling between theta phase (at 5Hz) and alpha/low-beta power, and **(C)** shows theta-dependent changes in functional connectivity, with greater Granger causal influence from mdPul to higher-order cortex (i.e., FEF and LIP) during the "good" theta phase (i.e., during periods of enhanced perceptual sensitivity). The black dots in **(B)** represent statistical significance after corrections for multiple comparisons.
